# Mud-Based Construction Material: Promising Properties of French Gravel Wash Mud Mixed with Byproducts, Seashells and Fly Ash as a Binder

**DOI:** 10.3390/ma14206216

**Published:** 2021-10-19

**Authors:** Yassine El Mendili, Manal Bouasria, Mohammed-Hichem Benzaama, Fouzia Khadraoui, Malo Le Guern, Daniel Chateigner, Stéphanie Gascoin, Jean-François Bardeau

**Affiliations:** 1 Laboratoire ESITC, COMUE Normandie Université, 1 Rue Pierre et Marie Curie, 14610 Epron, France; manal.bouasria@esitc-caen.fr (M.B.); mohammed-hichem.benzaama@esitc-caen.fr (M.-H.B.); fouzia.khadraoui-mehir@esitc-caen.fr (F.K.); malo.leguern@esitc-caen.fr (M.L.G.); 2 CRISMAT-ENSICAEN, UMR CNRS 6508, ENSICAEN, Université de Caen Normandie, 6 Boulevard Maréchal Juin, 14050 Caen, France; daniel.chateigner@ensicaen.fr (D.C.); stephanie.gascoin@ensicaen.fr (S.G.); 3 Institut des Molécules et Matériaux du Mans (IMMM), UMR CNRS 6283, Le Mans Université, Avenue Olivier Messiaen, 72085 Le Mans, France; Jean-Francois.Bardeau@univ-lemans.fr

**Keywords:** earth construction, gravel wash mud, byproducts, thermal properties, mechanical performance

## Abstract

The French gravel industry produces approximatively 6.5 million tons of gravel wash mud each year. This material offers very promising properties which require an in-depth characterization study before its use as a construction material, otherwise it is removed from value cycles by disposal in landfills. We examined the suitability of gravel wash mud and seashells, with fly ash as a binder, as an unfired earth construction material. Thermal and mechanical characterizations of the smart mixture composed of gravel wash mud, *Crepidula fornicata* shells and fly ash are performed. The new specimens exhibit high compressive strengths compared to usual earth construction materials, which appears as a good opportunity for a reduction in the thickness of walls. The use of fly ash and *Crepidula* shells in addition to gravel wash mud provides high silica and calcium contents, which both react with clay, leading to the formation of tobermorite and Al-tobermorite as a result of a pozzolanic reaction. Considering the reduction in porosity and improvements in strength, these new materials are good candidates to contribute significantly to the Sustainable Development Goals (SDGs) and reduce carbon emissions.

## 1. Introduction

Earth is one of the most ancient and extensively used building materials worldwide [1]. Historically, raw earth has been one of the main building materials in ancient cities such as Harappa (Pakistan), Duheros (España), Akhlet-Aton (Egypt), Jericho (Palestine), Chan-Chan (Peru), Atal-Huyuk (Turkey), Babylon (Iraq), Marrakech (Morocco) and others [1,2,3,4]. During the past centuries, overshadowed by the architecture of the fossil fuel consumption era, earth-based building materials are nowadays slowly regaining their status and becoming an integral part of sustainable construction [5]. In addition, earth has excellent abilities to maintain a more stable and higher indoor humidity level and thermal mass potential than the most commonly used building materials [6]. Earthen building construction is perhaps the most cost-effective solution to housing problems, with a limited demand on resources.

Mud is a mixture of fine-grained earth material and water. It offers significant sustainability as a building material [7,8,9,10]. One of the basic principles of sustainable construction practice is to use locally available materials, a characteristic of mud worldwide. Almost any type of mud can be used for construction once all the properties are tested and validated and the required ingredients added. Recently investigated mud materials include gravel wash muds (GWM) [11,12] and waste mud from gravel quarries. These refer to wet deposits that are generated during the extraction of gravel and the gravel aggregates in addition to sand washing. In France, the demand for aggregates reached 435 million tons in 2019 according to UNICEM, the national quarry industry and construction materials association [13], with 27% of this volume being concrete. Aggregates are used for the realization of civil engineering works, roads and buildings. The French gravel industry produces approximatively 6.5 million tons of gravel wash mud per year, according to UNICEM [13]. Due to its characteristics, this material could be used as a secondary raw material for various applications [11,12,14]. However, it is generally removed from recycling by disposal in landfills.

Zami and Lee [15] have shown that building with mud has many advantages, such as lower cost due to efficiency improvements and reduced energy and raw material consumption. In addition, by using locally available low-energy materials, the local economy is helped by increasing the labor cost and by generating new value. Mud is a mixture of multiple clay minerals whose structure is of upmost importance since they directly affect the durability and strength of the mud. The clay minerals are natural composites composed of layered hydrated alumino-silicates classed into four main groups, i.e., kaolin, illite, smectite and chlorite [16]. The smectite group is characterized by an extremely expandable crystal structure (upon hydration and heavy-element uptake), while illite, kaolin, chlorite and sepiolite are non-expandable clays. Expandable clays are subject to large volume variations (swelling and shrinking) upon contact with water [17]. Due to this, after water evaporation, drastic shrinkage and cracking can occur.

The use of byproducts with raw earth or GWM components can be considered a technically feasible green-like operation. Indeed, it does not require final combustion of the components, and this avoids the corresponding GHG emissions. Besides, it can solve the problem of shrinkage and cracking of clay minerals [18]. Consequently, by substituting traditional materials with such wastes, the building industry can achieve significant environmental benefits compared to the current situation [19].

The current research focuses on adapting earthen architecture to local and modern environments by using waste as an additive to earth for construction. For example, in Colombia, Villamizar et al. studied the effects of the addition of cassava peels and coal ash on the mechanical properties of compressed earth blocks [20]. They showed that coal ash led to the stabilization of the compressed blocks and that the cassava peels considerably improved the dry strength of the compressed blocks. In Brazil, Lima et al. were inspired to use sugarcane bagasse ash, resulting from industrial processes, as an additive to compressed earth blocks [21]. Their compressive strength and absorption test studies showed that such additives do not deteriorate the mechanical properties.

Thomas et al. proved that clay soil and oyster shell powder ground with rice husk ash were suitable as a binder for unfired mud bricks [22]. The conventional unfired bricks exhibit a larger compressive strength for 10% replacement of clayey soil by shell. The calcium carbonate from the oyster shells reacts with alumino-silicates and silicate-rich pozzolanic materials, leading to the formation of hydraulic products such as calcium silicate hydrates (CSHs) and calcium aluminate silicate hydrates (CASHs) [23,24,25]. The durability and the stability of these CSHs and CASHs depend on the structure of alumina and calcium siliceous phases, which influence their pozzolanicity and thus their mechanical performance.

Clayey earth soils undergo differential settlements, poor shear strength and high compressibility, and must be stabilized to enhance their mechanical performance. Chemical stabilization is a recently proposed technique to achieve this [26]. Fly ash (FA) stabilization is gaining more importance recently [27,28,29]. Indeed, FA has been extensively used as a binder and is a residue originating from coal-fired power plants. France generates around 3 million of FA annually [30]. The properties of FA, such as low permeability, low specific gravity and a high internal friction angle, lead to an increase in the earth bearing capacity and reduces its compressibility and settlement [27,29]. In addition, fly ash provides additional alumino-silicates brought into the system and thus amplifies the reactive surface for pozzolanic reaction [27,28]. These additional alumino-silicates can also react with calcium hydroxide originating from the hydration of *Crepidula* shells or clays [27,28,31].

Mud can be used either directly to construct walls or in the form of compressed mud blocks. The most popular mud or earth building techniques are rammed earth, daub, compressed earth blocks and cob [32]. In our region (Normandy, France), the most popular earth building technique is cob (Bauge in French), which uses a mixture of earth and fibers, such as straw [33].

In this paper, we study the suitability of gravel wash mud (GWM) and *Crepidula fornicata* shell powder, using fly ash as a binder, as a traditional cob material. The compressive strength and expanding properties of cob specimens based on GWM, CR and FA are investigated. Thermal properties and analysis of the formed hydrated products are also examined.

## 2. Materials and Techniques

### 2.1. Materials

#### 2.1.1. Gravel Wash Mud

The GWM was collected from a decantation basin of a gravel and sand company, operated by LafargeHolcim granulat (located at Yville-sur-Seine and Anneville-Ambourville in Normandy, France). The Lafarge gravel industry produces approximatively 200 tons of gravel wash mud at the Sablons quarry per year. The GWM water content is about 75% by weight. The GWM samples were dried for 6 h in an oven (at 105 °C) and then crushed into a fine powder. The use of GWM requires an in-depth characterization study before it is upgraded as a construction material.

#### 2.1.2. Fibers

It is known that fibers are added in earth construction as reinforcement and insulating materials. In this study, we use widely available and cheap wheat straws, which represent a low environmental impact. Additionally, only residual straws are used in order to prevent ILUC effects and keep their valorization with the largest renewability and CO_2_ emission neutrality as possible. The thermal conductivity of cheap wheat straw fibers is between 0.035 and 0.054 W·m^−1^·K^−1^ [33].

The wheat straws are provided by the local farmers (Laulne, Normandy). The physical properties of straw fibers are presented in Table 1.

#### 2.1.3. Fly Ash

We used a class F fly ash (FA) originating from coal-fired power plants, certified to an EN 450-1 standard with an absolute density of 2840 kg/m³ and a Blaine finesse of 3950 cm^2^/g. The FA byproducts were provided by the SURSCHISTE suppliers based in the north of France (Hornaing), producing approximatively 120,000 tons of FA per year. The used FA is a powder with spherical grains, which does not require further processing. Its chemical composition is given in Table 2.

**Figure 1:**
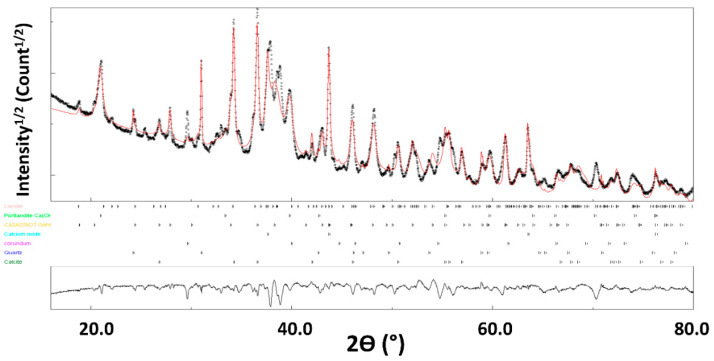
X-ray diffraction diagram of FA. The calculated diagram (red color) is superimposed onto the experimental profile (black color). At the bottom, we present the difference curve (I_exp_–I_calc_).

The XRD pattern of FA can reasonably refined (Figure 1) using 7 different phases (Table 3). The agreement factors R_wp_ = 6.8%, R_B_ = 5.9% and a goodness-of-fit below 2 (GoF = 1.7) both indicate the good reliability of our analyses. The microstrain values are also fitted during this step, and remain low for all the phases.

Quantitative phase analysis using Rietveld refinement indicates that the XRD pattern is indexed by the following major phases: larnite (51.2%), portlandite (16.9%), gehlenite (14.7%) and calcite (10.5%), with minor occurrence of quartz, calcium oxide and corundum.

#### 2.1.4. Crepidula Fornicata Shells

*Crepidula fornicate* (CR) are an invasive gastropod species causing problems in regard to halieutic resources due to its overgrowth on the coasts. For this work, the ground CR shells were provided by a fishing factory in Normandy. The crude shells were dried for 24 h in an oven at 105 °C and ground to powders in the ESITC laboratory through a 63-micron sieve. The specific gravity and the Blaine fineness were 2730 kg·m^−3^ and 8140 cm^2^·g^−1^. The elemental composition of CR powders was achieved by energy-dispersive X-ray spectroscopy (EDX) [34]. Calcium is the major cation. EDS also shows the presence of traces of Na, Si, Na, Mg, Al and S. In addition, X-ray diffraction and Raman analysis show aragonite as the main phase of *Crepidula* [34,35].

#### 2.1.5. Mix Design

Different mixes with various compositions were studied before reaching the optimal mixture (presented in this work), which exhibits the best mechanical and thermal performances.

The straw cuts, 5 cm long, were added randomly and mixed with the other components until the composite became homogeneous. The straw and the water contents were 2% and 18% by weight, respectively [36].

**Figure 2:**
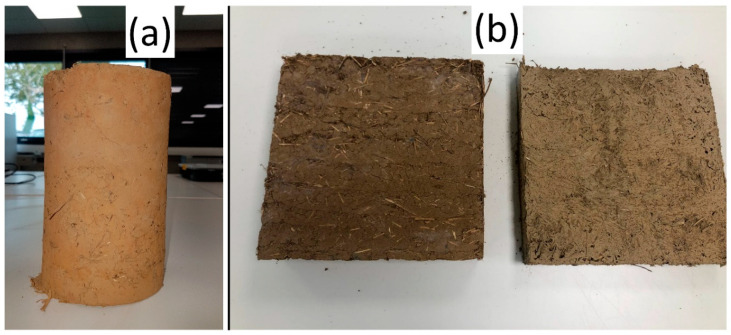
(**a**) Sample used for mechanical tests (Ø11 cm × 22 cm) and (**b**) samples used for thermal analysis (30 cm × 30 cm × 4 cm).

The optimized mix (Table 4) material (GWM-FA-CR) was placed into prismatic molds (30 cm × 30 cm × 4 cm) for thermal tests and into cylindrical molds (11 cm diameter and 22 cm height) for compressive tests (Figure 2).

After the GWM, FA and CR are mixed by hand and dry, water is added until the mixture becomes fluid. The cheap wheat straws are added gradually for 120 s. The GWM-FA-CR is then compacted manually and stored for 24 h at 20 ± 1 °C. After this period, the mix is then compacted manually in molds. The prepared molds are stored at room temperature for 48 h and then transferred for 48 h in an oven at 40 °C. The duration of drying is 3 weeks. After the drying process period, the mixture is kept under ambient conditions (20 °C and 50% relative humidity).

The samples are ready for characterization once equilibrium is reached (the mass difference is less than 0.1% between 2 daily weightings). Generally, the equilibrium is obtained after 48 h at room temperature (20 ± 1 °C).

The first observations show that no cracking is observed and that the shrinkage remains very low (<1%) for GWM-FA-CR after 3 weeks. The specific gravity of the specimen is around 1910 kg·m^−3^.

### 2.2. Experimental Techniques

Chemical analyses were performed by energy-dispersive X-ray spectroscopy (EDS) using a SUPRA™ 55 (SAPPHIRE; Carl Zeiss, Jena, Germany).

The Raman analyses were conducted using a DXR Thermo Scientific microscope (Wattam, MA, USA), employing a 532 nm laser source with a 2 mW laser with an integration time of 120 s and a nominal spectral resolution of 3 cm^−1^. The *Origin* software was used to fit the spectra. The mineralogical composition was achieved using the Raman Open Database [37].

X-ray diffraction analyses were recorded using a D8 Advance Vario 1 Bruker instrument equipped with pure-copper Kα radiation (λ = 1.54059 Å). X-ray data presented in this study are collected from 15° to 80° for 1 s per 0.01° step (with 2θ varying and 16 h per scan). The instrumental calibration is obtained by analyzing the LaB_6_ standard powder [38]. Quantification and crystalline phase identification were performed using the FPSM procedure (full-pattern search-match) and the COD database [39]. The diffraction patterns for the studied samples exhibit several phases. For the assignment of all the phases present in the XRD diagram, the elemental and chemical composition are primordial. For this purpose, the XRF and EDS analyses on the analyzed specimen were performed before. A rapid phase analysis using the online FPSM procedure: http://nanoair.dii.unitn.it:8080/sfpm/fpsmTest.html (accessed on 10 September 2021) was conducted to determine the probable phases as well as the lattice parameters for each phase. The COD database then provided the necessary information of crystal structures and the average size of each structure. The basis of the FPSM quantifications is the automatic identification of the first list of crystals. The complete list of phases is obtained using micro-Raman spectroscopy due to its larger sensitivity. Finally, a Rietveld quantification was performed using the MAUD software using the appropriate phases [40].

The thermogravimetric measurements were carried out using the NETZSCH instrument (STA 449 F5 Jupiter) on 60–80 mg of powders placed in an alumina pan with a heating rate of 10 °C/min from 20 °C to 900 °C in a Ar-flowing environment (50 mL/min).

Measurements of water vapor permeability were based on the wet cup method according to the standard NF EN ISO 12572 [41].

The dynamic vapor sorption (DVS) technique is used to investigate the solids’ interaction with vapor. In present study, sorption isotherms of the mixtures are studied according to the standard ISO 12571.

The thermal conductivity was conducted using a HFM (NETZSCH, Model HFM 436 Lambda) [26].

The specific heat capacity of a mixture is conducted according to the standard ISO 11357-4 [42] and using differential scanning calorimetry (NETZSCH, model STA 449 F3).

For the analyzes of specific surface area and the absolute density, we used the BET method [43] and a helium pycnometer (model Accupyc II 1340), respectively.

The mechanical resistance tests of the cylindrical GWM-FA-CR sample after 28 days were carried out by an electromechanical press of the IGM company. The compression tests were conducted by the application of an increasing load at constant speeds of 0.05 kN/s. The compressive strength is measured at 2% of shrinkage, which is representative of the earth wall behavior [26].

## 3. Results and Discussion

### 3.1. Physical and Chemical Properties of GWM

The GWM material contains mainly silicon, iron, calcium and aluminum for the major cations (Table 5), coherently with a clayey mud.

**Figure 3:**
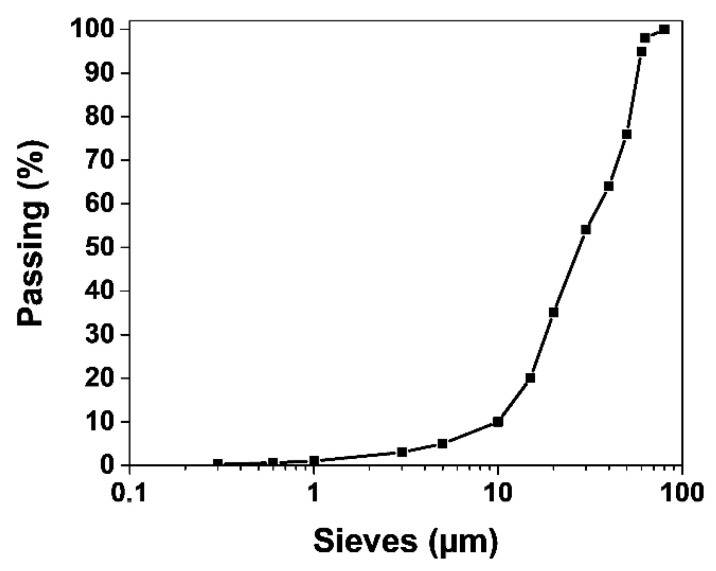
Particle size distribution of GWM powder.

The particle size distribution of GWM powder (Figure 3), measured using laser diffraction, exhibits d_10_ and d_90_ values of 3.67 and 46.33 μm, respectively. Particles with sizes less than 63 μm are about 98% of all particles.

**Figure 4:**
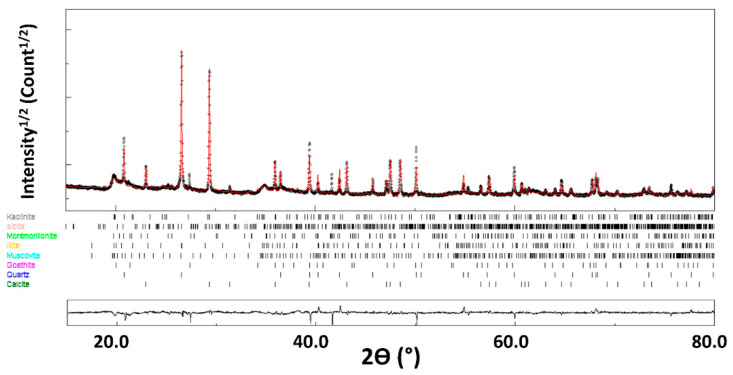
X-ray diffraction diagram of GWM. The calculated diagram (red color) is superimposed onto the experimental profile (black color). At the bottom, we present the difference curve (I_exp_–I_calc_).

While EDS analysis only provides element characterization, it can be important to know what crystalline phases these elements form in the material. For instance, Si atoms can be located in quartz and/or clays, two phases with very different behavior when incorporated into cob. The first phase analysis by the online FPSM procedure was launched. The FPSM uses a Rietveld fitting procedure to test all possible crystal structures from the COD database (restricted to the EDS-detected elements) which provides a ranked list of candidates for further quantification. The XRD diagram of the GWM powder is then fitted with the previous phase identification by FPSM. The XRD pattern of the GWM is reasonably refined (Figure 4) using eight different phases (Table 6). The agreement factors R_wp_ = 4.7%, R_B_ = 3.6% and a goodness-of-fit below 2 (GoF = 1.7) both indicate the good reliability of our analyses. The microstrain values are also fitted during this step, and remain low for all the phases.

Quantitative phase analysis using Rietveld refinement indicates that the XRD diagrams are indexed by the following major phases: calcite (30.8%), quartz (14.3%), montmorillonite (14.8%), illite (16.1%) and kaolinite (12.8%), with minor occurrence of albite, goethite and muscovite.

Muscovite is a hydrated phyllosilicate mineral containing aluminum and potassium [44]. Albite is a mineral of the feldspars family (silicate group, tectosilicate subgroup) with the formula NaAlSi_3_O_8_, which may contain traces of Ca, K and Mg [45]. Goethite is a mineral species, a variety of iron (III) oxyhydroxide and an α polymorph of the compound FeO(OH) [44]. Kaolinite is a mineral species composed of hydrated aluminum silicate and belongs to the subgroup of phyllosilicates [44].

Illite is the name of a group of non-swelling clay minerals. Illites are composed of three layers of phyllosilicates—one layer of aluminum (Al) surrounded by two layers of silicate (Si). They are structurally very close to micas (muscovite, biotite) and other silicates (feldspar, feldspathoids, orthosis and others), from which they are produced by bisiallitization, a reaction that takes place when water is attacked under certain temperature and pressure conditions [44]. Montmorillonite is a mineral composed of aluminum silicate and hydrated magnesium. Montmorillonite is a 2:1-type clay, also called TOT (tetrahedron/octahedron/tetrahedron). This means that a montmorillonite sheet is formed of three layers: an octahedral layer of Al(OH^−^)_5_O and two SiO_4_ tetrahedral layers [44]. One of the most remarkable properties of montmorillonites is their swelling capacity, resulting from the entry of water into the space between the layers. Montmorillonite dispersed in water very easily gives a stable, colloidal suspension. On the other hand, this aptitude for swelling and conversely for shrinkage (collapse of the clay layers during desiccation) poses significant problems from a geotechnical point of view, causing sometimes-significant displacements at the foundations according to variations in humidity of the sub-soil.

Some clays have the ability to increase their interfoliar spaces. This property comes from the incorporation of hydrated cations (Na, Ca, etc.) making it possible to compensate for permanent charge deficits. This phenomenon no longer exists if the clay charge is too high (e.g., micas or muskovite in our sample: total clay charge of −1 perfectly counterbalanced by the dehydrated cations (K)) or zero (e.g., pyrophyllite, talc: total clay charge of 0, no interfoliar cation). The expandable species are those whose charge varies from 0.3 to 0.8, which includes the subclass of smectites. It is the water incorporated via the hydrated cations which allows the swelling of the crystalline structure. The swelling is all the more important as the humidity is high. The only expandable species present in our GWM is montmorillonite, with a rate of 14.8%. The presence of muscovite, illite, albite and kaolinite in the GWM specimen will influence the shrinkage behavior. These crystals contain small quantities of water and they have a low intercrystalline swelling behavior [46].

### 3.2. Characterization of GWM-FA-CR

#### 3.2.1. Compressive Strength of GWM-FA-CR

The results also show that GWM-FA-CR produced larger strengths after 3 weeks of curing compared to those of standard cob materials (Table 7). GWMs are subject to differential settlement and low shear strength, and require additional stabilization to enhance their properties. The use of FA as a stabilizer is one of the proposed solutions. This waste is gaining more importance recently [27,28,29]. Indeed, FA has been extensively used as a binder. The properties of FA such as low permeability, low specific gravity and a high internal friction angle lead to an increase in the earth bearing capacity and reduces its compressibility and settlement [27,29]. In addition, fly ash provides additional calcium silicates and alumino-silicates brought into the system, and thus amplifies the reactive surface for a pozzolanic reaction [27,28]. These additional alumino-silicates can also react with calcium hydroxide originating from hydration of Crepidula shells or clays [27,28,31]. The formation of calcium silicate and alumino-silicate hydrate products will be confirmed in the next XRD and Raman section.

#### 3.2.2. Analyses of GWM-FA-CR Sample Using XRD

**Figure 5:**
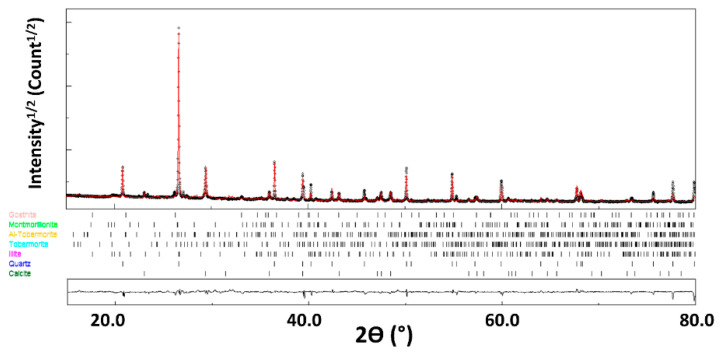
X-ray diffraction diagram of GWM-FA-CR. The calculated diagram (red color) is superimposed onto the experimental profile (black color). At the bottom, we present the difference curve (I_exp_–I_calc_).

An XRD pattern of the GWM-FA-CR sample is refined using seven main phases (Figure 5 and Table 8). The agreement factors are: R_wp_ = 7.8% and R_B_ = 9.7% (GoF = 1.55).

Quantitative phase analysis indicates that the XRD lines of the GWM-FA-CR sample are dominated by calcite, quartz, tobermorite and Al-tobermorite. In addition to these phases, montmorillonite, illite and goethite originating from GWM are also detected in lower amounts. Consequently, part of these phases did not react, neither with FA nor with CR. The calcium originating from CR and GWM reacts with alumino-silicates from GWM and silicate-rich FA pozzolanic materials, which leads to the formation of hydrated phases, mainly calcium aluminate silicate (Al-tobermorite) and calcium silicate (Al-tobermorite) hydrates. Tobermorite minerals exhibit orthorhombic symmetry and a basal spacing of 11 Å with the formula Ca_4+x_(Al_y_Si_6–y_)O_15+2x–y_∙5 H_2_O, where x and y are from 0 to 1 [47]. Tobermorite has a high cation exchange capacity and high selectivity towards Al [48]. The Al-substituted tobermorite is considered to be a new family of selective cation exchangers [48]. The substitution of Si by Al occurs in the bridging and non-bridging tetrahedron sites [49,50]. The synthesized Al-substituted forms of tobermorite are formed in the presence of some raw materials, such as FA [51,52] and/or phases from the CaO-SiO_2_-H_2_O system [53].

#### 3.2.3. Analyses of GWM and GWM-FA-CR Sample by DSC

**Figure 6:**
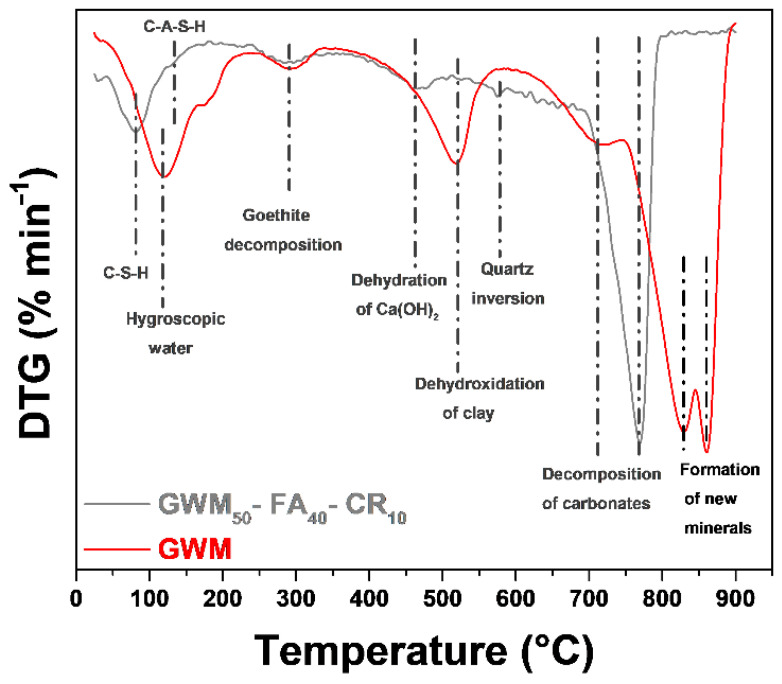
DTG curves of the GWM sample and GWM-FA-CR sample after 28 days of curing time.

Figure 6 shows the derivative thermogravimetry calorimetry (DTG) skeletons of the GWM and GWM-FA-CR samples. The mass loss between 100 and 130 °C is attributed to the dehydration and evaporation of free water. The DTG peak present in the GWM-FA-CR from 85 to 100 °C is assigned to the evaporation of free water and the dehydration of CSH gel [54]. The other peak located at 130 °C indicates a dehydration of CASH-type reaction products [55], and the peak above 460 °C the dehydration of portlandite [56].

For the GWM sample, the first endothermic peak, which begins at 110 °C, corresponds with the removal of hygroscopic water between the clay particles [57].

The mass loss between 500 and 560 °C is attributed to the dehydration of the clay minerals [58,59]. For GWM-FA-CR, we can observe that the clay’s dehydration peaks are very low compared to those of the GWM sample. This phenomenon is due to the reactivity of these clay minerals with FA and CR to form CSH and CASH hydrated products. The mass loss at 295 °C is attributed to the decomposition of hydroxides, such as goethite [60]. A discrete endothermic peak around 575 °C can also be observed. This peak is attributed to the allotropic transformation of α into β-quartz [61].

The endothermic peaks at 720 °C, with a large range in the DSC curve, are due to the decomposition of calcium carbonates [62]. It is important to note that these endothermic peaks are strongly asymmetric, which indicates that the decomposition kinetic increases with temperature until the depletion of carbonates. The carbonate decomposition leads to CO_2_ release to the atmosphere.

The exothermic DTA peaks above 800 °C, present only in the GWM sample, are associated with the decomposition of clay minerals. They form a new mineral with a spinel-type structure [63].

#### 3.2.4. Raman Spectroscopy Analyses

**Figure 7:**
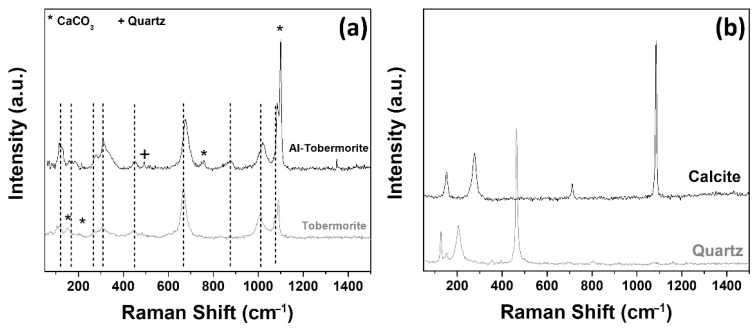
Raman spectra of the phases detected in the GMW-FA-CR mixture after 28 days of curing: (**a**) CSH and CASH, (**b**) calcite and quartz.

The Raman analyses of the hydrated structures formed in the GWM-FA-CR after 28 days show the presence of tobermorite and Al-tobermorite (Figure 7). In addition to the CSH and CSAH phases, the Raman spectra also show the presence of quartz as well as calcite [64,65]. It is important to note that no trace of aragonite was detected. This is probably due to the complete dissolution of this phase. Indeed, the solubility (LogK_sp_) of aragonite in water at 25 °C is −8.336 ± 0.020, leading to the formation of Ca^2+^ and CO_3_^2−^ ions [66]. Therefore, the dissolved Ca^2+^ ions will participate in the hydration reactions and then in the formation of the CSH and CASH phases.

The nature of the hydrated CSH and CSAH structures have been largely studied by Raman spectroscopy [67,68]. All Raman spectra of tobermorite show the presence of the most intense vibration mode attributed to the Si–O stretching vibration at 665 cm^−1^ (Figure 7a). The band near 440 cm^−1^ is attributed to the Si–O–Si twisting and stretching modes. The vibration modes attributed to the lattice vibrations of Ca-O polyhedra are present at low-wavenumber zones (<350 cm^−1^). The vibration modes in the wavenumber region between 850 and 1100 cm^−1^ are assigned to the Q_n_ symmetric stretching modes of silicate Si-O.

The Raman spectra show that the Q_2_ symmetric stretching mode present at 1005–1020 cm^−1^ in the GWM-FA-CR specimen split into two vibration bands at 1007 and 1016 cm^−1^. This splitting is the signature of the Si–O–Si chain length modification by a change in the amount of silica tetrahedron Q_2p_ (pairing) and Q_2b_ (bridging) [69]. The split of Q_2_ is mainly due to a high quantity of fly ash silicon. Indeed, the silicates, brought by FA, react with the calcium originating from CR and GWM to form additional CSH gel as a result of a pozzolanic reaction, and thus lead to a reduction in porosity and enhanced strength. The Raman spectra show also the presence of the C-O stretching mode in the CaCO_3_ group at 1085 cm^−1^.

For Al-tobermorite, we can observe that the wavenumbers of the vibration modes are red-shifted compared to those of pure tobermorite. This phenomenon is due to Al substitution in tobermorite. Al substitution in tobermorite induces a decrease in the cell parameters compared to those of tobermorite (Table 8). The Al substitution leads to an increase in the (002) interplanar crystal spacing due to the larger radius of Al^3+^ compared to Si^4+^ [70]. The substitution of Al enhanced the degree of the silicate chain polymerization in tobermorite [70].

The main hydration products responsible for the increase in mechanical performance properties are: tobermorite and alumino-silicate hydrates (Al-tobermorite).

#### 3.2.5. Thermal Conductivity of GMW-FA-CR

A thermal conductivity (λ) lowered to two-third the one of the standard cob is also observed for GMW-FA-CR (Table 9), with 0.58 W·m^−1^·K^−1^ and 0.35 W·m^−1^·K^−1^, respectively. This shows that the FA-CR addition improves the thermal insulation character of the GMW construction material.

This behavior is peculiar because the density of the standard cob is smaller than the one of GMW-FA-CR. In this latter, the greater density results in a smaller thermal conductivity, a sign of significant modifications of the thermal carriers in the material with the introduction of FA and CR. Our measurements cannot dissociate between the contribution of electrons and phonons to thermal conductivity, but the former contribution is generally more affected by density than the latter. In such low-electronic-conduction materials, the former can be expected to mainly contribute.

#### 3.2.6. Moisture Sorption Isotherm of GMW-FA-CR

**Figure 8:**
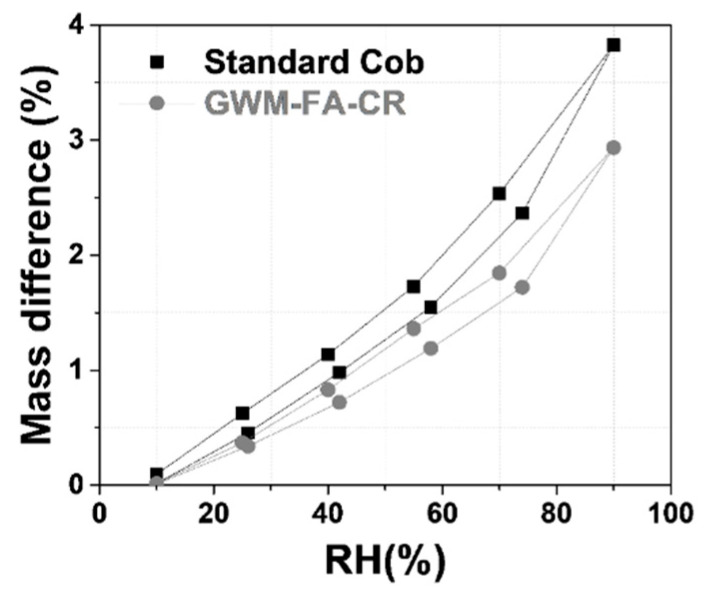
Moisture sorption isotherm of the GWM-FA-CR mix and a cob material at room temperature.

The curve shapes are similar for GMW-FA-CR and the cob specimens, and correspond to a sigmoid (Figure 8). The moisture sorption curves belong to type II isotherms [73]. An increase in the mass difference from the GWM-FA-CR to the cob specimen is noticed. This increase could be attributed to two main factors: porosity and differences in phase fractions (quantity of clay minerals) in the cob and the GMW-FA-CR specimen [74]. In the GWM-FA-CR, the addition of FA and aragonite originating from CR on the GWM lead to a decrease in clayey minerals. In addition, the porosity of the GWM-FA-CR mix is only two-third that of the standard cob (Table 10), further decreasing the mass difference.

#### 3.2.7. Specific Heat Capacity of GWM-FA-CR

**Figure 9:**
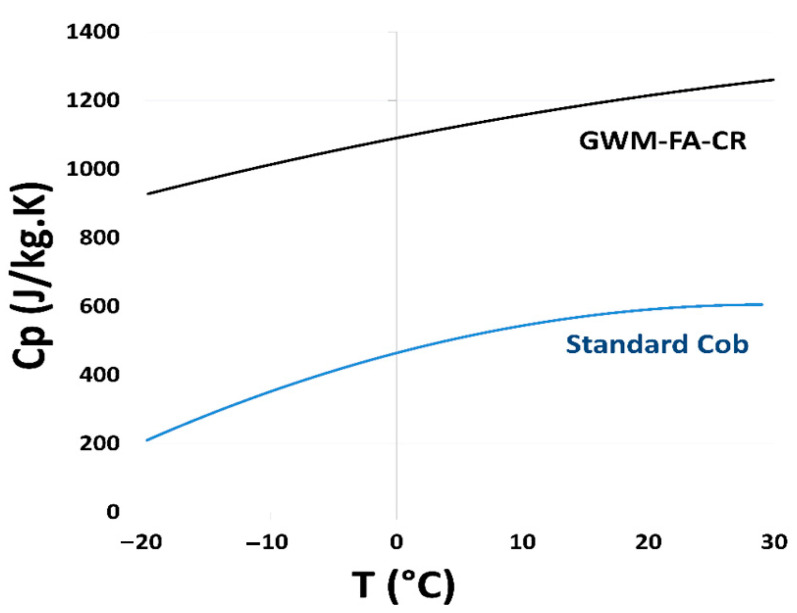
The variation in the specific heat capacity of the GWM-FA-CR mix and a standard cob material between −20 °C and 30 °C.

The specific heat capacity values for the GWM-FA-CR mix are between 925 and 1250 J·kg^−1^·K^−1^. These values are very high compared to those of the standard cob specimen at all temperatures. (Figure 9). For the construction sector, building with materials with a high specific heat capacity performance is very important in regard to the energy aspect. In addition, the specific thermal capacity of materials is very important in the construction industry for the evaluation of the indoor comfort of the building’s occupants. In summer, walls with a high thermal capacity keep rooms cool for a long time. In winter, they retain heat in buildings longer.

#### 3.2.8. Thermal Insulating Wall with GWM-FA-CR

**Figure 10:**
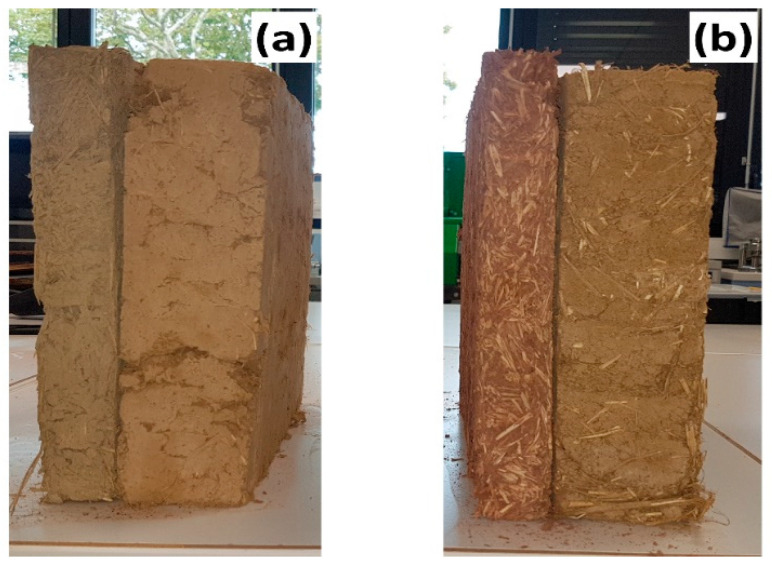
Cob walls with two layers: (**a**) GWM-FA-CR mixture and (**b**) usual cob.

There is a strong tradition for cob constructions in Germany [75]. This system of construction is made with a secondary layer. The first layer is composed of traditional cob as a structural wall and the second layer, based on light earth, is used as a thermal insulating wall (Figure 10).

For this purpose, we investigated the performance of the thermal and mechanical performance of a thermal insulating material based on a GWM-FA-CR mixture incorporating reed fibers (length between 4 and 6 cm). According to the usual method in bibliography [33], the reed content used was 25%.

The thermal conductivity of the light-earth wall based on GWM-FA-CR is 0.112 W·m^−1^·K^−1^ at 20 °C (Table 11). For the standard cob insulating specimens, λ is 0.157 W·m^−1^·K^−1^ at 20 °C. This showed that the use of GWM-FA-CR lead to a decrease in thermal conductivity compared to light-earth material. In addition, we show that this GWM-FA-CR mixture enhanced the compressive strength of the earth insulating materials. This result is surprising and shows that we can use this GWM-FA-CR mixture for both structural and insulating walls. In terms of performance criteria, the suitability of GWM for earth construction without further additives was not possible due to the presence of swelling clay.

It is important to note that different GWM powders from 12 various quarries in Normandy have been studied by EDS, XRF and X-ray, and the quantitative analysis shows that the crystalline phases identified were quartz, calcite and muscovite, kaolinite and illite as clay minerals followed by iron oxy-hydroxides (Table 12). However, a rapid strength was observed and the maximal compressive strength achieved after 3 weeks of drying was around 5 MPa for all the specimens made with GMW, 25% of FA and 5% of CR. Furthermore, with this addition, the content of swelling clays is reduced whatever the origin of GWM.

## 4. Conclusions

This study presents the effect of the use of a GMW-FNS-CR mix as an earth construction material. The thermal and mechanical characterizations of the GWM-FA-CR sample as well as the microstructural properties yield some encouraging results, which are highlighted as follows:

1. The use of GWM, CR and FA can greatly contribute to the Sustainable Development Goals (SDGs) and to reducing carbon emissions.
2. The formation of tobermorite and Al-tobermorite leads to high mechanical performance properties of the GWM specimen.
3. The increase in compressive strength compared to usual cob materials results in a reduction in the cob wall thickness, and therefore a gain in the quantities of materials used in cob construction.
4. The use of FA and CR provides two advantages:
  (1) The high silica and calcium contents originating from FA and CR, respectively, react with clays and lead to the formation of tobermorite and Al-tobermorite as a result of a pozzolanic reaction, and thus lead to a reduction in porosity and enhanced strength.
  (2) The thermal conductivity of the GMW-FA-CR is reduced and the specific heat capacity is enhanced compared to those of the usual cob construction materials used in Normandy.

## Figures and Tables

**Table 1:** Properties of Our Wheat Straw Fibers.

Diameter (mm)	Length (cm)	Density (Kg·m^−3^)	Initial Water Content (%)	Tensile Strength (MPa)
1–4	10–58	1910 ± 5	10.7	23.9 ± 3.5

**Table 2:** Chemical Composition of Fly Ash in Weight.% Obtained by Energy-Dispersive X-ray Spectroscopy.

Component	SiO_2_	CaO	Fe_2_O_3_	Al_2_O_3_	K_2_O	MgO	TiO_2_	SO_3_	Na_2_O	MnO_2_	P_2_O_5_	Cl
wt.%	53.3	5.1	8.5	23.6	3.0	3.0	1.0	1.1	0.6	0.5	0.2	0.1

**Table 3:** The Mineralogical Composition of FA.

Phases	COD Reference	V (%)	Lattice Type + Space Group	Lattice Parameters (Å)	⟨D⟩ (nm)	⟨ε^2^⟩^1/2^
Calcite CaCO_3_	1547347	10.5 (3)	Trigonal R-3c:H	a = 4991 c = 17,101	77 (5)	1 × 10^−4^
Quartz SiO_2_	1526860	2.7 (2)	Trigonal P3_2_21	a = 4919 c = 5408	362 (20)	1 × 10^−4^
Corundum Al_2_O_3_	1000017	1.1 (2)	Trigonal R-3c:H	a = 4704 c = 13,653	135 (5)	1 × 10^−4^
Calcium oxide CaO	1000044	2.9 (2)	Cubic Fm-3m	a = 4807	138 (5)	1 × 10^−4^
Gehlenite Ca_2_Al_2_SiO_7_	1000048	14.7 (4)	Tetragonal P-421m	a = 7714 c = 5062	66 (2)	2 × 10^−4^
Portlandite Ca(OH)_2_	1001768	16.9 (3)	Trigonal R-3c:H	a = 3595 c = 4914	55 (2)	6 × 10^−4^
Larnite Ca_2_SiO_4_	9012789	51.2 (2)	Monoclinic P121/n1	a = 5436 b = 6769 c = 9356 β = 94,172	97 (2)	6 × 10^−4^

**Table 4:** Composition of the GMW-FA-CR Mix.

Mix	GWM	FA	CR	Fiber	Water
Mass (%)	50	25	5	2	18

**Table 5:** Physical and chemical properties of GWM.

Element	%
SiO_2_	61.3
Al_2_O_3_	9.8
Fe_2_O_3_	10.2
CaO	10.1
MgO	2.9
Na_2_O	0.1
K_2_O	1.8
SO_3_	<0.1
P_2_O_5_	<0.1
Cl^−^	0.11
**Physical and Hygrothermal Properties**	-
Specific surface (in cm^2^·g^−1^)	4120
Absolute density (in kg·m^−3^)	925
Thermal conductivity (in W·m^−1^·K^−1^)	0.935 ± 0.01
Water vapor permeability (in kg·m^−1^·s^−1^·Pa^−1^)	2.4 × 10^−11^

**Table 6:** The Mineralogical Composition of GWM.

Phases	COD Reference	V (%)	Lattice Type + Space Group	Lattice Parameters (Å)	⟨D⟩ (nm)	⟨ε^2^⟩^1/2^
Calcite CaCO_3_	1547347	30.8 (3)	Trigonal R-3c:H	a = 4987 c = 17,056	392 (20)	8 × 10^−4^
Quartz SiO_2_	1526860	14.3 (2)	Trigonal P3_2_21	a = 4915 c = 5407	219 (10)	4 × 10^−4^
Albite NaAlSiO_3_O_8_	1556999	2.3 (2)	Triclinic P1	a = 8166 b = 12845 c = 7188 α = 94,240 β = 116,590 γ = 87,715	30 (5)	6 × 10^−3^
Kaolinite Al_2_Si_2_O_5_(OH)_4_	1011045	12.8 (2)	Monoclinic Cc:b1	a = 5246 b = 8886 c = 14,672 β = 100,565	26 (5)	6 × 10^−4^
Illite (K,H_3_O)(Al,Mg,Fe)_2_(Si,Al)_4_O_10_[(OH)_2_,(H_2_O)]	2300190	16.1 (4)	Monoclinic C2/m:b1	a = 5171 b = 8942 c = 10,229 β = 100,683 (1)	70 (5)	2 × 10^−4^
Goethite α-FeO(OH)	2211652	2.4 (3)	Orthorhombic Pbnm:cab	a = 4579 b = 9945 c = 2998	21 (1)	6 × 10^−4^
Montmorillonite (Na,Ca)_0.3_ (Al,Mg)_2_Si_4_O_10_(OH)_2_	1100106	14.8 (2)	Monoclinic C2/c:b1	a = 5451 b = 9067 c = 10,255 β = 100,780	125 (4)	6 ×10^−4^
Muscovite KAl_2_(AlSi_3_O_10_)(F,OH)_2_	1100011	6.7 (4)	Monoclinic C2/c:b1	a = 5183 b = 9006 c = 20,186 β = 95,702	89 (5)	2 × 10^−3^

**Table 7:** Compressive strength of the cob and the GWM-FA-CR specimens after 3 weeks of curing compared to those of standard cob building materials from the literature.

Sample	Compressive Strength (Mpa)
GWM-FA-CR	5.38 ± 0.23
Standard cob	2.03 ± 0.02
Cob materials from the literature [33]	1.42–1.52

**Table 8:** The mineralogical composition of GWM-FA-CR after 3 weeks of curing.

Phases	COD Reference	V (%)	Lattice Type + Space Group	Lattice Parameters (Å)	⟨D⟩ (nm)	⟨ε^2^⟩^1/2^
Calcite CaCO_3_	1547347	26.3 (5)	Trigonal R-3c:H	a = 4984 c = 17,047	308 (20)	8 × 10^−4^
Quartz SiO_2_	1526860	22.3 (3)	Trigonal P3_2_21	a = 4913 c = 5404	690 (10)	5 × 10^−4^
Montmorillonite (Na,Ca)_0.3_ (Al,Mg)_2_Si_4_O_10_(OH)_2_	1100106	6.4 (2)	Monoclinic C2/c:b1	a = 5441 b = 9003 c = 10,250 β = 100,323	66 (4)	6 × 10^−4^
Illite (K,H_3_O)(Al,Mg,Fe)_2_(Si,Al)_4_O_10_[(OH)_2_,(H_2_O)]	2300190	7.1 (3)	Monoclinic C2/m:b1	a = 5183 b = 8986 c = 10,171 β = 100,50	66 (5)	1 × 10^−4^
Tobermorite Ca_5_Si_6_O_16_(OH)_2_·4H_2_O	9005498	19.5 (3)	Monoclinic Cm:c2	a = 6806 b = 7402 c = 22,390 γ = 124,345	58 (1)	5 × 10^−4^
Al-tobermorite Al_0.5_Ca_4.9_H_10.7_O_22_Si_5.5_	1527001	15.6 (3)	Monoclinic Cm:c2	a = 6770 b = 7359 c = 22,227 γ = 123,770	43 (4)	6 × 10^−2^
Goethite α-FeO(OH)	2211652	2.6 (3)	Orthorhombic Pbnm:cab	a = 4588 b = 10,093 c = 2984	19 (1)	6 × 10^−4^

**Table 9:** Thermal Conductivity of GWM-FA-CR Specimen and the Standard Cob Compared to Those of Cob Building Materials from the Literature.

Sample	Density (kg·m^−3^)	Thermal Conductivity (W·m^−1^·K^−1^)
GMW-FA-CR	1910 ± 5	0.35 ± 0.03
Standard cob	1843 ± 5	0.58 ± 0.02
Cob materials from the literature [33,36,70,71,72]	1200–2000	0.47–0.93

**Table 10:** Porosity Measured on the Cob Material and GWM-FA-CR Mix.

Sample	Standard Cob	GMW-FA-CR
**Porosity**	61%	42%

**Table 11:** Physical Properties of the Thermal Insulating Materials Based on GWM-FA-CR and 25% Reed Fiber after 28 Days of Curing Compared to Those of Standard Cob Insulating Building Materials from the Literature.

Sample	Compressive Strength (MPa)	Thermal Conductivity (W·m^−1^·K^−1^)
GMW-FA-CR + 25% reed fiber	0.140 ± 0.01	0.112 ± 0.005
Standard cob insulating wall [26]	0.079 ± 0.01	0.157 ± 0.005

**Table 12:** Chemical and Mineralogical Properties of Different GWM Specimens.

Chemical Analyses (in Weight.%)	Mineralogy
SiO_2_: from 41 to 78%	Quartz: from 10 to 25%
Al_2_O_3_: from 6 to 15%	Kaolinite: from 8 to 20%
Fe_2_O_3_: from 5 to 12.5% CaO: from 3 to 16%	Calcite: from 23 to 35% Illite: from 11 to 19%
MgO: from 0.6 to 3.6%	Smectites: from 10 to 28%
Na_2_O < 0.2%	Feldspaths: from 0 to 5%
K_2_O: from 1 to 2.1%	Plagioclases: from 0 to 1%
SO_3_ < 0.1	Iron oxy-hydroxides: from 1 to 6%
P_2_O_5_ < 0.1	Micas: from 2 to 9%
Cl^−^ < 0.2%	**Swelling clays** **: from 9 to 18%**

## Data Availability

The data presented in the present paper are available from the corresponding author upon a reasonable request.
